# Supplementary material for: Deletion of wfs1 Impairs Oligodendrocyte Precursor Cells Dorsal Distribution and Myelination Through the wfs1-hmgcs1 Axis in Zebrafish
Source: Biology (Basel). 2026 Aug 16;15(16):1402. doi: 10.3390/biology15161402 (PMC13510151; doi:10.3390/biology15161402)
Supplement: Supplementary file 1 [file biology-15-01402-s001.zip › File S1-Transcriptome sequencing data and differentially expressed genes (DEGs) in wfs1-- zebrafish at 5 dpf/1.GO/wfs1KOvsWT/GSEA/wfs1KO/SNARE_COMPLEX/SNARE_COMPLEX(GO_0031201).html]

Details for gene set SNARE\_COMPLEX(GO:0031201)[GSEA]


|  || Dataset | deg.sample |
| Phenotype | sample.cls |
| Upregulated in class | wfs1KO |
| GeneSet | SNARE\_COMPLEX(GO:0031201) |
| Enrichment Score (ES) | 0.4538706 |
| Normalized Enrichment Score (NES) | 1.8439397 |
| Nominal p-value | 0.0 |
| FDR q-value | 0.103262745 |
| FWER p-Value | 0.563 |
Table: GSEA Results Summary

  

Fig 1: Enrichment plot: SNARE\_COMPLEX(GO:0031201)
  
Profile of the Running ES Score & Positions of GeneSet Members on the Rank Ordered List

  

| PROBE | DESCRIPTION   (from dataset) | GENE SYMBOL | GENE\_TITLE | RANK IN GENE LIST | RANK METRIC SCORE | RUNNING ES | CORE ENRICHMENT || 1 | 436605 | stx5a |  |  | 32 | 4.929 | 0.1252 | Yes |
| 2 | 449923 | si:ch73-234b20.5 |  |  | 327 | 3.546 | 0.2067 | Yes |
| 3 | 571872 | zgc:165520 |  |  | 964 | 2.142 | 0.2416 | Yes |
| 4 | 393236 | sybl1 |  |  | 967 | 2.136 | 0.2962 | Yes |
| 5 | 393836 | stx11b.1 |  |  | 1443 | 1.695 | 0.3246 | Yes |
| 6 | 325484 | vamp5 |  |  | 1642 | 1.574 | 0.3587 | Yes |
| 7 | 445167 | stx2a |  |  | 1898 | 1.414 | 0.3869 | Yes |
| 8 | 327007 | vamp8 |  |  | 2179 | 1.294 | 0.4112 | Yes |
| 9 | 393346 | stx8 |  |  | 2411 | 1.204 | 0.4348 | Yes |
| 10 | 553682 | napba |  |  | 2932 | 1.054 | 0.4454 | Yes |
| 11 | 492808 | stx17 |  |  | 3434 | 0.945 | 0.4539 | Yes |
| 12 | 415135 | bet1 |  |  | 6204 | 0.568 | 0.3814 | No |
| 13 | 100006625 | stx1a |  |  | 6986 | 0.493 | 0.3695 | No |
| 14 | 795732 | bnip1b |  |  | 9195 | 0.353 | 0.3092 | No |
| 15 | 569124 | stx12l |  |  | 9356 | 0.344 | 0.3129 | No |
| 16 | 751727 | snap47 |  |  | 9894 | 0.310 | 0.3040 | No |
| 17 | 393159 | snap23.1 |  |  | 10097 | 0.296 | 0.3052 | No |
| 18 | 562857 | stx16 |  |  | 12041 | 0.188 | 0.2490 | No |
| 19 | 556701 | cplx2 |  |  | 12214 | 0.179 | 0.2482 | No |
| 20 | 393515 | stx3a |  |  | 12741 | 0.152 | 0.2355 | No |
| 21 | 114464 | sec22ba |  |  | 12870 | 0.147 | 0.2353 | No |
| 22 | 323735 | stx4 |  |  | 13648 | 0.109 | 0.2137 | No |
| 23 | 568195 | napbb |  |  | 13725 | 0.106 | 0.2140 | No |
| 24 | 393708 | vamp4 |  |  | 14550 | 0.067 | 0.1898 | No |
| 25 | 553460 | snap29 |  |  | 14882 | 0.052 | 0.1807 | No |
| 26 | 30712 | snap25a |  |  | 14927 | 0.050 | 0.1806 | No |
| 27 | 562669 | stx7l |  |  | 16112 | -0.006 | 0.1436 | No |
| 28 | 415141 | stx12 |  |  | 16442 | -0.016 | 0.1336 | No |
| 29 | 323105 | stx5al |  |  | 16849 | -0.028 | 0.1216 | No |
| 30 | 30711 | snap25b |  |  | 17054 | -0.035 | 0.1161 | No |
| 31 | 571925 | stxbp5b |  |  | 17240 | -0.042 | 0.1113 | No |
| 32 | 445493 | bet1l |  |  | 17624 | -0.059 | 0.1008 | No |
| 33 | 436805 | zgc:92912 |  |  | 17804 | -0.068 | 0.0969 | No |
| 34 | 100004361 | cplx4c |  |  | 18550 | -0.101 | 0.0761 | No |
| 35 | 560648 | si:ch211-81a5.8 |  |  | 19141 | -0.128 | 0.0609 | No |
| 36 | 436742 | napgb |  |  | 19226 | -0.132 | 0.0616 | No |
| 37 | 406707 | vamp1 |  |  | 19580 | -0.147 | 0.0543 | No |
| 38 | 336281 | vamp2 |  |  | 19711 | -0.153 | 0.0541 | No |
| 39 | 797490 | cplx4b |  |  | 19954 | -0.164 | 0.0507 | No |
| 40 | 415163 | vamp3 |  |  | 20257 | -0.177 | 0.0458 | No |
| 41 | 567306 | napaa |  |  | 20833 | -0.206 | 0.0330 | No |
| 42 | 58038 | stx1b |  |  | 22861 | -0.317 | -0.0226 | No |
| 43 | 327082 | napab |  |  | 23096 | -0.334 | -0.0214 | No |
| 44 | 794623 | cplx3b |  |  | 23961 | -0.389 | -0.0386 | No |
| 45 | 556533 | cplx3a |  |  | 24357 | -0.415 | -0.0404 | No |
| 46 | 554130 | stx18 |  |  | 24476 | -0.418 | -0.0334 | No |
| 47 | 324569 | gosr2 |  |  | 24481 | -0.419 | -0.0228 | No |
| 48 | 447892 | zgc:101731 |  |  | 25359 | -0.472 | -0.0383 | No |
| 49 | 101882035 | si:ch211-208k15.1 |  |  | 25607 | -0.494 | -0.0334 | No |
| 50 | 100005065 | stx11b.2 |  |  | 25648 | -0.498 | -0.0219 | No |
| 51 | 641321 | snap23.2 |  |  | 25829 | -0.513 | -0.0144 | No |
| 52 | 327277 | sec22bb |  |  | 26082 | -0.536 | -0.0086 | No |
| 53 | 541353 | napga |  |  | 26083 | -0.536 | 0.0051 | No |
| 54 | 100536636 | stx2b |  |  | 26197 | -0.547 | 0.0156 | No |
| 55 | 405846 | stx11a |  |  | 26408 | -0.568 | 0.0235 | No |
| 56 | 567385 | vti1a |  |  | 26783 | -0.607 | 0.0273 | No |
| 57 | 565822 | bnip1a |  |  | 26966 | -0.626 | 0.0376 | No |
| 58 | 768157 | cplx4a |  |  | 27063 | -0.636 | 0.0509 | No |
| 59 | 436732 | cplx2l |  |  | 27358 | -0.655 | 0.0584 | No |
| 60 | 394011 | vti1b |  |  | 28249 | -0.778 | 0.0504 | No |
| 61 | 556789 | stxbp5a |  |  | 29403 | -1.032 | 0.0405 | No |
| 62 | 393294 | use1 |  |  | 30434 | -1.467 | 0.0457 | No |
Table: GSEA details
[plain text format]

  

Fig 2: SNARE\_COMPLEX(GO:0031201)
  
Blue-Pink O' Gram in the Space of the Analyzed GeneSet

  

Fig 3: SNARE\_COMPLEX(GO:0031201): Random ES distribution
  
Gene set null distribution of ES for
 **SNARE\_COMPLEX(GO:0031201)**
